# External evaluation and self-monitoring of the Baby-friendly Hospital Initiative’s maternity hospitals in Brazil

**DOI:** 10.1186/s13006-018-0195-4

**Published:** 2019-01-05

**Authors:** Renara Guedes Araújo, Vânia de Matos Fonseca, Maria Inês Couto de Oliveira, Eloane Gonçalves Ramos

**Affiliations:** 1 Coordination of the Brazilian Child Health and Breastfeeding Program, Anexo do Ministério da Saúde, SAF Sul Quadra 1, Bloco B, 4 andar, Brasília, DF CEP 70058-900 Brazil; 2 Fernandes Figueira Institute, Oswaldo Cruz Foundation, Unidade de Pesquisa Clínica, Av. Rui Barbosa, 716, Rio de Janeiro, RJ CEP 20021-140 Brazil; 3 Biostatistics and Epidemiology Department, Public Health Institute, Fluminense Federal University, Av. Marquês do Paraná, 303, Niterói, RJ CEP 24020-071 Brazil

**Keywords:** Breastfeeding, System, Monitoring, Program evaluation, Maternity hospital, Normative evaluation, Baby-friendly hospital initiative

## Abstract

**Background:**

In Brazil, the Baby-Friendly Hospital Initiative (BFHI) proposes following the criteria, the “Ten Steps to Successful Breastfeeding”, International Code of Marketing of Breast-milk Substitutes and Good birth and delivery practices. Brazilian Baby-Friendly Hospitals are reassessed triennially by external evaluators and annually by self-monitoring. This study aimed to verify if the self-monitoring system fulfills its role of enabling accredited hospitals to assess and improve their compliance with the BFHI criteria. In this sense, we will analyze the self-monitoring evaluation results and compare them with those of the external reassessment.

**Methods:**

This descriptive evaluation study of the compliance with the BFHI criteria by the Brazilian Baby-Friendly Hospitals by self-monitoring evaluators from 2010 to 2015 and by external evaluators in 2015.

**Results:**

Self-monitoring was performed in all years from 2010 to 2015 by 143 BFHI accredited hospitals. The trend of the levels of compliance with BFHI’s criteria according to self-monitoring evaluations was stable over the assessed period. Most criteria presented compliance above 70%, except Step 4 (skin-to-skin contact and breastfeeding in the first hour of life), with mean compliance of 67%. Steps 1 (written policy), 7 (rooming-in) and 9 (give no artificial teats) showed mean compliance above 90%. Regarding the external evaluation carried out in 2015, the criteria with lowest compliance were Step 4 and Woman-Friendly care, both below 50%. Steps 9 and 10 (refer mothers to breastfeeding support groups) reached levels of compliance above 90%. For 2015, self-monitoring provided significant higher compliance levels than those from external evaluations in most criteria, except Step 3 (prenatal information on breastfeeding) and Step 10. There was a difference of more than 30% points between evaluations of Steps 1 (written policy), 2 (training), 5 (show mothers how to breastfeed), Woman-Friendly Care and father or mother stay with their newborn.

**Conclusions:**

The self-monitoring system fulfilled partially its role of allowing accredited hospitals to self-assess and improve rates of compliance with BFHI criteria. Future trainings of hospital managers need to address difficulties and identify solutions to improve implementation of Steps 4 and 6.

## Background

In 1991, the Baby-Friendly Hospital Initiative (BFHI) was launched by the World Health Organization (WHO) and the United Nations Children’s Fund (UNICEF) to increase breastfeeding rates [1, 2]. Maternity hospitals were encouraged to implement the “Ten Steps to Successful Breastfeeding” and the International Code of Marketing of Breast-milk Substitutes. External evaluators assess maternity hospitals practice of these Steps and compliance with the International Code. Those that accomplish these standards are accredited as Baby-Friendly Hospitals. All BFHI facilities should be re-assessed at least every three to five years [3].

Based on data compiled from 168 countries, the overall coverage of Baby-Friendly Hospitals is 10%, with regional variations of 36% in the European region and less than 5% in Africa and South-East Asia [3]. In Brazil 10% of the country maternity hospitals is accredited in the BFHI [4], a total of 326 maternities. One-third of births of the country take place at Baby-Friendly Hospitals in 2015 [5].

In Brazil, since 1981 a National Breastfeeding Incentive Program had been launched, with mass media campaigns and social mobilization to increase breastfeeding practice in the country [6]. In 1988, Brazil implemented the Unified Health System, with universal coverage, administered by the Ministry of Health and organized in a regionalized, hierarchical and decentralized network. Health actions and services are offered at all federative levels: Union, States and Municipalities.

The Baby-Friendly Hospital Initiative was adopted in 1992, including a certification process and a triennial reassessment by external assessors [7], and is also coordinated by the Ministry of Health and included within the decentralized structure of the Unified Health System. External evaluators are health professionals trained in a 40 h course accredited as BFHI evaluators by the Ministry of Health. Two evaluators who cannot be linked to the evaluated hospital visit the facilities and verify the compliance of their practices with the BFHI criteria [8]. This external evaluation allows the Ministry of Health to judge whether the criteria are being met and to decide if the BFHI title should be maintained.

From 2010, BFHI-accredited hospitals started to monitor themselves annually by self-evaluation. Self-monitoring evaluators are health professionals from the staff, generally members of the Hospital Breastfeeding Committee, that use a web-based database and monitoring system [9]. This system was adapted from an application created by UNICEF and WHO in 2007, with financial and human resources investment by the Ministry of Health for its development and training of BFHI assessors to multiply the use of this system in their home states [10].

After 16 years of BFHI in Brazil, the Second Survey of Breastfeeding Prevalence, carried out in 2008 found that the children born in BFHI accredited hospitals had a median length of exclusive breastfeeding of 60.2 days and those born in non-accredited hospitals of 48.1 days. Births in Baby-Friendly Hospitals increased by 9% the likelihood of breastfeeding within the first hour of life [11].

From 2015, in order to be accredited by the BFHI in Brazil, a hospital must meet the internationally established global criteria, which include the Ten Steps to Successful Breastfeeding and, additionally, the Brazilian version of the International Code of Marketing of Breastmilk Substitutes (BCode), and Good birth and delivery practices, which include Parents stay with the newborn admitted to the neonatal unit (PWN) and Woman-Friendly care (WFC) [12].

Studies performed in BFHI-accredited hospitals in Brazil [13], the Caribbean and Latin America identified difficulties related to the maintenance of the title [14]. Then it was proposed that the external reassessment of these hospitals occurred every three to five years, more frequently than in most of the surveyed countries, to maintain its quality standard [14]. However, only half of the countries with active BFHI have established a re-evaluation process, most of which with a frequency lower than every five years [3].

Annual self-monitoring was introduced to assess if the hospital continues to comply with the BFHI criteria and allows the hospital staff to verify which Steps must be improved in order to maintain the quality standard required by the BFHI.

This study aimed to verify if the self-monitoring system fulfills its role of enabling accredited hospitals to self-assess and improve their compliance with the BFHI criteria. In this sense, we will analyze the self-monitoring evaluation results and compare them with those of the external reassessment.

## Methods

This is a descriptive study of evaluation of the compliance with the BFHI criteria by the Brazilian Baby-Friendly Hospitals according to the self-monitoring and external evaluation of these hospitals.

Data from the self and external evaluations previously recorded in the database system available at the Brazilian government website were used [15]. Public access to this database is restricted. For this research, permission for use was granted by the Ministry of Health.

**Fig. 1:**
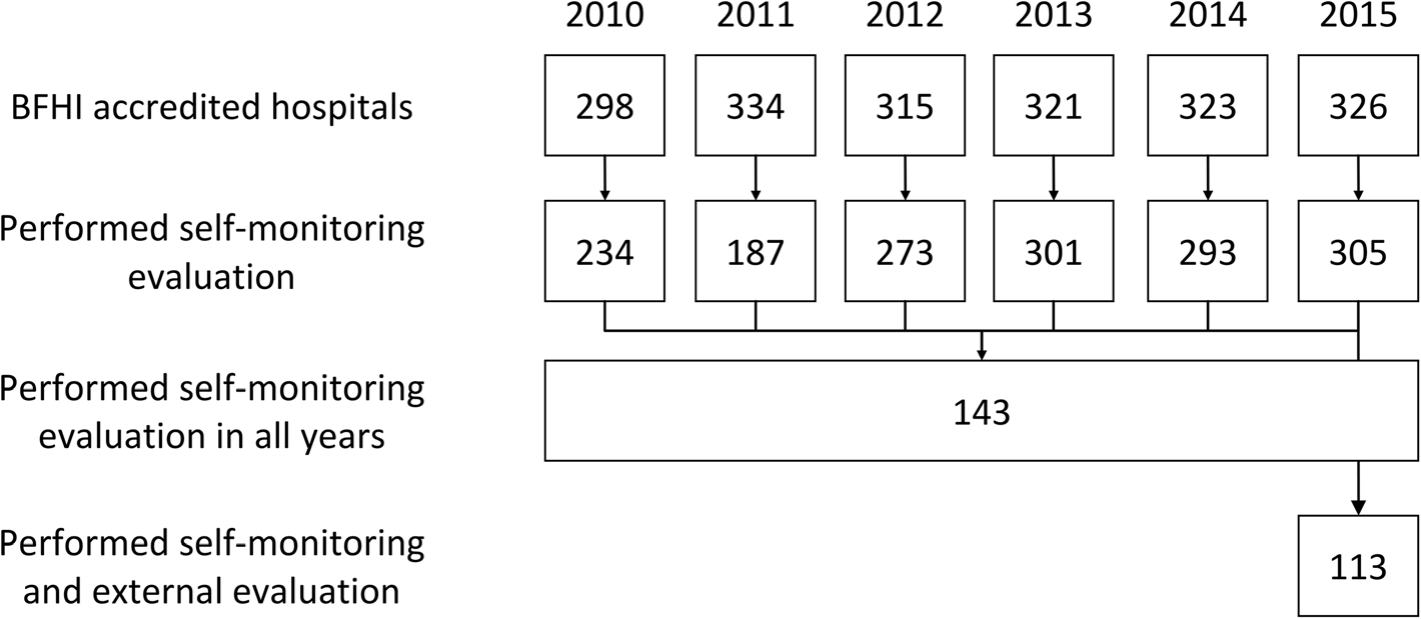
Numbers of BFHI-accredited hospitals in each phase of the study

We included all accredited Baby-Friendly Hospitals which performed self-monitoring evaluation annually between 2010 and 2015 inclusive and also included those which received external evaluation in 2015 (Fig. 1).

We have made two analyses of the compliance rates with individual phases – one over a six-year period of internal self-monitoring and a second analysis comparing the results between the self-monitoring and external evaluation in 2015.

Both the self-monitoring and external evaluations measure the compliance with each of the BFHI criteria: Ten Steps to Successful Breastfeeding, BCode (Brazilian version of the International Code of Marketing of Breastmilk Substitutes), PWN (Parents With Newborn) and WFC (Woman-Friendly Care), which are described in Table 1 [16]. To accomplish that, in each maternity hospital the evaluators perform:

1. Interview with the director of the hospital or responsible for the maternity hospital, analysis of hospital data, printed materials on BFHI rules and routines and observations made on the maternity hospital, prenatal care and birth/delivery service. These questions and observations are about Steps 1 and 2, BCode and WFC criteria.
2. Interviews with clinical and non-clinical staff members, pregnant women, rooming-in mothers and with infants in neonatal intensive care units. These questions are about Steps 3 to 10 and PWN criteria.
3. Based on the interviews and observations, the evaluator must indicate in a summary report the compliance with each criterion. A criterion is considered complied when 80% of health professionals, pregnant women or mothers follow it. It depends on to whom the criterion is intended.

**Table 1:** Criteria for certification on the Baby-Friendly Hospital Initiative

Ten Steps to Successful Breastfeeding	
Step 1	Have a written breastfeeding policy that is routinely communicated to all health care staff.
Step 2	Train all health care staff in skills necessary to implement this policy.
Step 3	Inform all pregnant women about the benefits and management of breastfeeding.
Step 4	Facilitate immediate and uninterrupted skin-to-skin contact and support mothers to initiate breastfeeding within an hour of birth.
Step 5	Show mothers how to breastfeed, and how to maintain lactation even if they should be separated from their infants.
Step 6	Give newborn infants no food or drink other than breast milk, unless medically indicated.
Step 7	Practice rooming-in - that is, allow mothers and infants to remain together - 24 h a day.
Step 8	Encourage breastfeeding on demand.
Step 9	Give no artificial teats or pacifiers (also called dummies or soothers) to breastfeeding infants.
Step 10	Foster the establishment of breastfeeding support groups and refer mothers to them on discharge from the hospital or clinic
BCode	Brazilian Code of Marketing of Breastmilk Substitutes. Comply with Law 11,265 of January 3, 2006 and the Brazilian version of the International Code of Marketing of Breastmilk Substitutes
Good birth and delivery practices	
PWN	Parents with Newborn. Ensure free access to the mother and father and the permanence of the mother or father 24 h a day with the newborn admitted to the neonatal unit.
WFC	Women-Friendly Care. Comply with the Global Criteria Woman-Friendly Care, which includes encouraging companions of the women choice, allowing women to drink and eat light foods during labor, encouraging women to consider the use of non-drug methods of pain relief, walk and move about during labor, assume positions of their choice while giving birth, care that does not involve invasive procedures such as rupture of the membranes, episiotomies, acceleration or induction of labor, instrumental deliveries, or caesarean sections unless specifically required for a complication and the reason is explained to the mother.

Interviews are conducted using standardized questionnaires and the summary data is reported in the online system.

For our analysis, we used the compliance with each Step to Successful Breastfeeding, BCode, PWN, and WFC (Table 1), entered in the summary report of each maternity hospital.

The results of the analysis of the self-monitoring evaluations from 2010 to 2015 were described by levels of compliance, stated as percentages of hospitals that complied with each criterion, overall and by year. We computed the proportion of compliance as the mean of a set of ones (hospital complied with the criterion) and zeros (hospital did not comply with the criterion). Then the 95% CI was calculated as 1.96* SE (the standard error of the mean), where SE = SD/√n and SD = standard deviation. The analysis comparing self-monitoring and external evaluations performed in 2015 used the MacNemar test to verify differences between categorical evaluations of two paired samples. A significance level of 5% was used, as usual in most studies, indicating the Type I error we are willing to accept: the probability of rejecting the null hypothesis giving it is true. The analyses were performed in software SPSS 17 and R.

## Results

From 2010 and 2015 the number of BFHI-accredited Brazilian hospitals that performed the self-monitoring evaluation varied from 187 to 305 (Fig. 1). From these, 143 performed the self-monitoring in all the years of the study period and were included in the self-evaluation analysis. For the comparison with the external evaluation, only the 113 hospitals that had also been submitted to this evaluation in 2015 were included (Fig. 1). As Good birth and delivery practices criteria PWN and WFC were introduced in 2014, these criteria were analyzed only in 2014 and 2015.

**Fig. 2:**
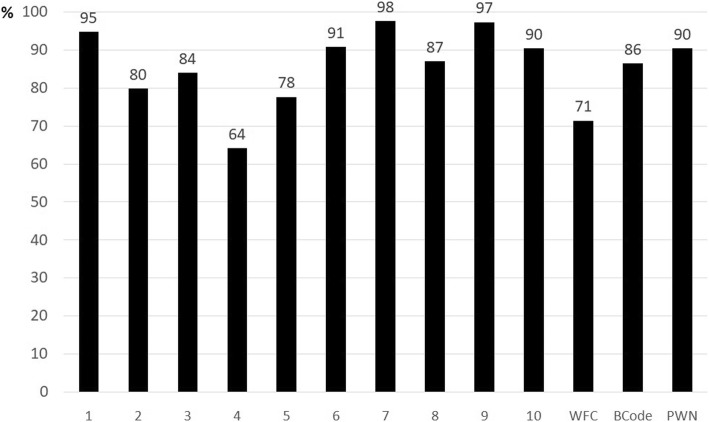
Mean percentage of compliance with BFHI criteria. Brazil 2010–2015. Legend: Mean percentage of compliance with the Ten Steps and other criteria by Baby-Friendly Hospitals, according to self-monitoring in the period 2010–2015. Brazil (*n* = 143). Footnote: 1–10 = Ten Steps to Successful Breastfeeding; BCode = Brazilian Code of Marketing of Breastmilk Substitutes; WFC = Woman-Friendly Care; PWN = Father or mother stay with the Newborn in neonatal unit

The mean percentages of compliance of hospital’s practice (*n* = 143) with the BFHI’s criteria in the period 2010–2015, according to self-monitoring evaluation, are depicted in Fig. 2. Compliance was greater than 90% in Step 1 (written policy on breastfeeding), Step 6 (not giving the newborn drink or food other than breast milk), Step 7 (not giving artificial teats or pacifiers to newborns) and Step 10 (refer mothers to post-discharge breastfeeding support groups). Step 4 (helping mothers to start breastfeeding in the first hour of life) had the lowest compliance (64.1%). Regarding good practices of delivery and birth, the PWN had the highest level of compliance (Fig. 2).

**Fig. 3:**
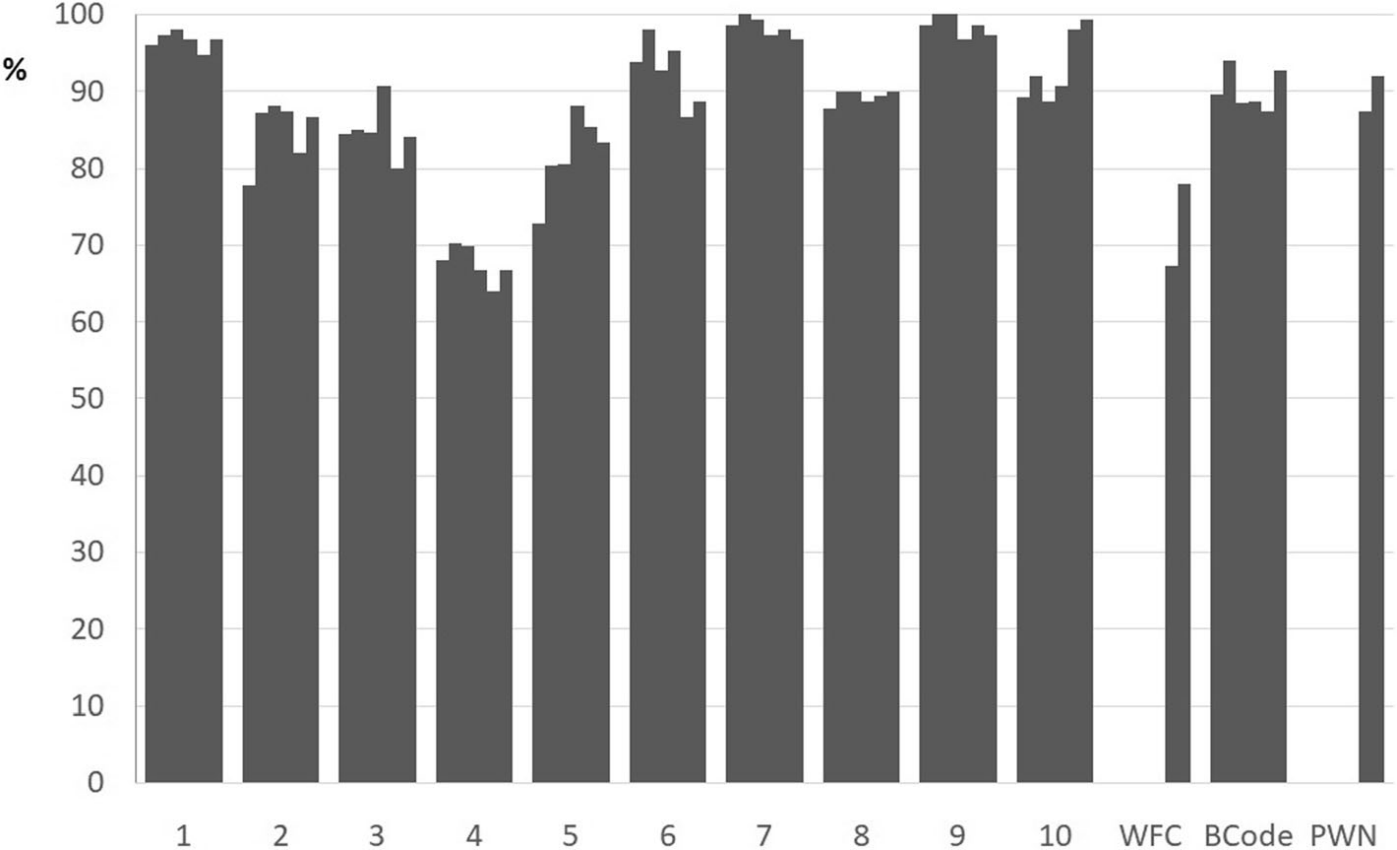
Percentage of compliance with BFHI criteria by year in the period 2010–2015. Brazil. Legend: Temporal evolution of the percentage of compliance with the Ten Steps and other criteria by Baby-Friendly Hospitals, according to self-monitoring in the period 2010–2015. Brazil (*n* = 143). Footnote: 1–10 = Ten Steps to Successful Breastfeeding; BCode = Brazilian Code of Marketing of Breastmilk Substitutes; WFC = Woman-Friendly Care; PWN = Father or mother stay with the Newborn

The year by year temporal evolution of the hospitals (*n* = 143) based on self-monitoring compliance with BFHI’s criteria from 2010 to 2015 are depicted in Fig. 3. We see that the criteria with a level of compliance of more than 90% in all years of the historical series were Steps 1, 7 and 9, while Step 4 had the lowest level of compliance. There was a relative stability in the implementation of Steps 1, 2, 3, 7, 8, 9 and BCode throughout the period, while Steps 5 and 10, PNW and WFC showed an increasing level of compliance, especially WFC (Fig. 3). Steps 4 and 6 showed a decreasing trend.

**Fig. 4:**
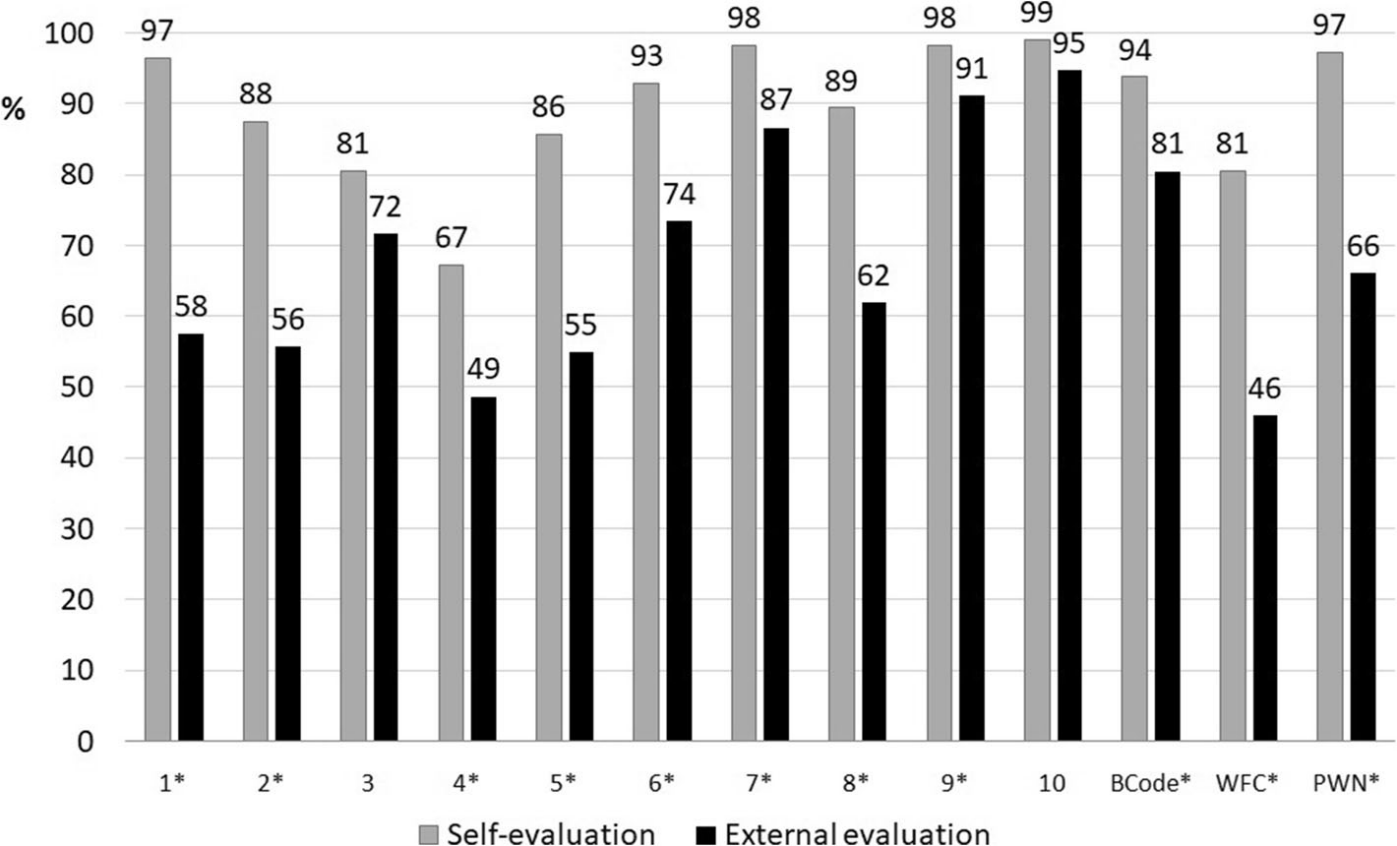
Comparison of compliance with BFHI criteria by self-monitoring and external evaluation. Brazil 2015. Legend: Comparison of compliance with the Ten Steps and other criteria by Baby-Friendly Hospitals that carried out self-monitoring and external evaluation in 2015. Brazil (*n* = 113). Footnote: 1–10 = Ten Steps to Successful Breastfeeding; BCode = Brazilian Code of Marketing of Breastmilk Substitutes; WFC = Woman-Friendly Care; PWN = Father or mother stay with the Newborn; * Criteria that had a significant difference (Mc Nemar test *p* - value < 0.05) between self and external evaluations

Regarding the external evaluation carried out in 2015, we can see in the dark bars of Fig. 4 that the criteria with lowest compliance were Step 4 and WFC, both below 50%, while Steps 9 and 10 had a level of compliance above 90%.

When comparing the self-monitoring carried out in 2015 with the external evaluation carried out in the same year (Fig. 4), we found that self-monitoring produced higher compliance levels than external evaluation. Confidence intervals and *p* - values are shown in Table 2. All criteria showed statistically significant differences, except Steps 3 and 10. There was a large discrepancy in the evaluation of Steps 1 (written policy), 2 (training), 5 (show mothers how to breastfeed), WFC and PWN, with a difference of more than 30% points between both evaluations (Fig. 4).

**Table 2:** Comparison of the compliance with the Ten Steps to Successful Breastfeeding and other criteria by Baby-Friendly Hospitals by self-monitoring and external evaluation in 2015. Brazil (*n* = 113)

Steps and criteria	Self-monitoring		External evaluation		*p* - value
	% compliance	95% CI	% compliance	95% CI	
Step 1 (written policy)	96	93, 100	58	49, 67	< 0.01
Step 2 (staff training)	88	81, 94	56	46, 65	< 0.01
Step 3 (prenatal information on breastfeeding)	81	73, 88	72	63, 80	0.076
Step 4 (skin-to-skin contact and breastfeeding in the first hour of life)	67	58, 76	49	39, 58	0.004
Step 5 (breastfeeding management)	86	79, 92	55	46, 65	< 0.01
Step 6 (give no food or drink other than breast milk)	93	88, 98	73	65, 82	< 0.01
Step 7 (practice rooming-in)	98	96, 101	87	80, 93	< 0.01
Step 8 (breastfeeding on demand)	89	83, 95	62	52, 71	< 0.01
Step 9 (give no artificial teats or pacifiers)	98	96, 101	91	57, 75	0.039
Step 10 (refer mothers to breastfeeding support groups on discharge from the hospital)	99	97, 101	95	90, 99	0.063
BCode	94	89, 98	81	73, 88	< 0.01
WFC	81	73, 88	46	37, 56	< 0.01
PWN	97	93, 100	66	57, 75	< 0.01

Values of *p* are from McNemar test. The level of significance is 5%. CI = Confidence Interval; BCode = Brazilian Code of Marketing of Breastmilk Substitutes; WFC = Woman-Friendly Care; PWN = Father or mother stay with the Newborn admitted to the neonatal unit

The criteria with more than 70% compliance according to both modalities of evaluation were Steps 3 (guidance on advantages and management of breastfeeding in prenatal care), 6, 7, 9 and 10, as well as BCode (Fig. 4). Step 4 had the lowest level of compliance according to both evaluation modalities.

## Discussion

Self-monitoring identified that the Brazilian Baby-Friendly Hospitals have consistently complied with most of the criteria in the period evaluated. This aspect can be considered a positive sign of sustained quality, especially for those with a high percentage of compliance. On the other hand, it was expected that, over the years, the criteria with lower level of compliance would show a positive trend, since internal monitoring is a tool to help hospitals improve their performance throughout the process. These expected improvement in compliance with Steps 4 and 6 did not occur, probably because the increasing cesarean rates in Brazil may be hampering these practices [17].

Self-monitoring evaluations provided more favorable results than external evaluation, when comparing self-monitoring and external evaluations carried out in 2015. External evaluators are neutral because they have no relationship with the hospital and are highly trained to perform this evaluation. Negative aspects should be pointed out in relation to the self-evaluators, such as being part of the structure to be evaluated, possibly inhibiting the expression of opinions by respondents and having difficulty in expressing value judgments, tending to minimize the elements that may demonstrate failure in the activities evaluated [18].

So, we cannot assure that self-monitoring can mirror the situation of each hospital in relation to the Ten Steps and additional criteria in the same way as external evaluation does. Nevertheless, both evaluations agreed that Step 4 had the lowest level of compliance and Steps 10, 9 and 7 achieved the best level of compliance (Fig. 4). Both self-evaluation and external evaluation identified the best and worst performing Steps. Thus, the aim of help the staff of the hospital in identifying the procedures to be improved was at least partially obtained.

In the international setting, the BFHI has also been facing numerous challenges for its sustainability and financing [14, 19, 20]. The financing hardships exist both at the government level and internally at maternity hospitals, hampering assessments and the training of employees of Baby-Friendly Hospitals [3]. Many countries have failed to establish effective re-evaluation mechanisms. Most of them do not have self-monitoring systems to ensure that BFHI quality standards are maintained [21].

Studies conducted in Australia [19], Croatia [20] and some Latin American countries [14] have shown that BFHI’s sustainability depends on several factors. Emphasis is placed on the importance of a robust national breastfeeding policy, with active national and local breastfeeding committees, financial incentive, involvement of hospital managers, articulation between federal and state governments, and the involvement of all sectors of society to support hospitals that wish to be part of this initiative and maintain their title. Brazil has a strong national breastfeeding policy, internationally recognized [6]. Periodic self-monitoring and external reassessment of hospitals have been strategic tools for maintaining the quality of Brazilian Baby-Friendly Hospitals [14, 19, 20].

Although the BFHI is undergoing an international crisis [14], where several countries discuss its sustainability, in Brazil, the number of accredited Baby-Friendly Hospitals increased from 298 to 334 in the period 2010–2015 (Fig. 1). Since the system began to be used, external evaluators have participated in training and updating workshops. During these workshops, the importance of the role of the external evaluator as a supporter of the qualified hospitals is emphasized. It is recommended that this professional will assist hospitals in staff training, as well as clarify issues regarding all Steps and criteria of the BFHI [16]. The external evaluator is also a link between the hospital, state and municipality, an important factor for the proper implementation of the BFHI. The articulation between the federated entities is paramount for the success of this initiative [8]. The role of states and municipalities is also fundamental in supporting hospitals that are qualified to maintain the title of Baby-Friendly Hospitals, especially regarding the formation of adequate human resources to conduct self-monitoring and involve hospital managers.

### Levels of compliance with individual criteria

Step 4, regarding skin-to-skin contact and breastfeeding in the first hour of life, showed the greatest compliance difficulty. In a study conducted in 2009, when the BFHI implementation in Rio de Janeiro was evaluated, it was also observed that only 57% of the Baby-Friendly Hospitals practiced breastfeeding in the first hour of life, although 86.5% practiced skin-to-skin contact [13]. Similar data were found in the evaluation of 32 priority maternity hospitals of *Rede Cegonha* (a Brazilian Ministry of Health strategy to improve mother and child care), with 24 Baby-Friendly Hospitals in 2013, when about half of these maternity facilities performed well on skin-to-skin contact and breastfeeding in the first hour of life [22].

Difficulties in complying with Step 4 were also observed in the international setting. A similar result was found in a study that evaluated the implementation of BFHI in Croatia, from 27 questionnaires for self-assessment of maternity hospitals, less than 50% complied with Step 4 [20]. In Québec, Canada, at nine Baby-Friendly Hospitals evaluated, compliance with Step 4 was 53% [23].

Studies show that compliance with Step 4 relies on several factors, one of which is the continuous training of health staff [24], allowing mothers to be guided in the delivery room regarding the baby’s latch and ability to suck. In an analysis of BFHI’s situation in 26 countries in Latin America and the Caribbean, the main obstacles to compliance with Step 4 were the lack of qualification of professionals to help mothers who started breastfeeding, and hospital routines related to newborns [14]. Other factors have been associated with delayed initiation of breastfeeding, such as lack of prenatal care, cesarean delivery, lack of knowledge of the Human Immunodeficiency Virus (HIV) serological status at delivery [25], and lack of hospital staff listening to mothers’ breastfeeding concerns [26]. Brazil has a very high cesarean rate (56.6% in 2013) [27], which may be contributing to the difficulty in complying with Step 4, due to postoperative care routines that delay skin-to-skin contact and breastfeeding in the postpartum period. Regarding the lack of knowledge about HIV serological status, in the prenatal period, the rapid HIV test is provided at admission for delivery, but it is not always it is done swiftly, and when the result is available only after birth, breastfeeding can be improperly postponed [28].

Although Step 4 is not satisfactorily complied with by the Brazilian Baby-Friendly Hospitals, being born in a BFHI accredited hospital is a protective factor for breastfeeding at birth. The Born in Brazil Survey, conducted between 2011 and 2012 in 266 hospitals in the five macro-regions of the country, showed that 56% of the children were breastfed in the first hour of life, and this proportion was 69.4% between those born in Baby-Friendly Hospitals, and 47.7% in non-accredited hospitals. Prenatal care in the public network, vaginal delivery and full term newborns were also factors that were positively associated with breastfeeding in the first hour of life [5].

The most accomplished Steps were 7 (rooming-in), 9 (not giving artificial teats or pacifiers to newborns) and 10 (breastfeeding support on discharge from hospital). In 1983, the Ministry of Health approved standards for the implementation of the rooming-in system in hospitals [29], which may have contributed to a consolidation of this legislation in the country. Concerning Step 9, its compliance may be related to the insertion of Brazilian Code compliance, which since 2004 prohibits the advertising and donation of teats, pacifiers and bottles, as a BFHI criterion since 2004 [30]. Compliance with Step 10 was 95% or above in both evaluations, indicating that the post discharge breastfeeding support network has expanded throughout the national territory, composed basically of primary healthcare facilities. In 2008, The Ministry of Health launched a PHC-targeted strategy, namely, the *Rede Amamenta Brasil* (Brazilian Breastfeeding Network), which recently incorporated the promotion of healthy complementary food and started to be denominated as the Brazilian Breastfeeding and Feeding Strategy, and is expanding throughout the national territory [31].

The Step that draws attention to the gap in the level of compliance between the two evaluations is Step 1, which concerns the hospital having a policy, a written standard for breastfeeding routinely transmitted to the entire health staff. This possible overestimation of compliance by the self-evaluation may be because self-evaluators do not understand what a written policy is, sometimes considering that affixing the Ten Steps to the success of breastfeeding on hospital walls corresponds to compliance with this criterion [8].

Regarding the additional criteria, the evaluation of the BCode showed in both evaluations a high degree of compliance. The large difference of more than 30 percentage points between assessments of Woman-Friendly Care (WFC) and Parents stay with the newborn admitted to the neonatal unit (PWN) occurred possibly because these are recent criteria, for which self-evaluators are not yet able to assess accurately. Possibly, the municipality responsible for this action, as per the Ordinance [32], has not yet carried out training on the new Brazilian BFHI criteria.

However, self-monitoring indicates that Woman-Friendly Care, included as a criterion for accreditation in the BFHI in Brazil since 2014, showed a positive trend in its level of compliance from 2014 to 2015. One factor that may have contributed to this result was the fact that the *Rede Cegonha*, a strategy that advocates good birth and delivery practices, has been implemented in the country since 2011 [33]. A study in 32 maternity hospitals of the *Rede Cegonha*, where practices related to the Woman-Friendly Care were observed (such as the provision of non-pharmacological methods for pain relief, birthing in the non-supine position, supply of liquids during labor and presence of a doula during labor), pointed out that less than a third of maternity hospitals (27%) performed well [22]. Self-monitoring indicated that parents staying with newborn (PWN), another criterion included in Brazil in 2014, had a high level of compliance in that year and in 2015. Contributed to this finding the existence, since 2012, of Ordinance 930 of the Ministry of Health, which already provided for the inclusion of parents or caregivers of the baby, remaining with their child 24 h with free access, issues that are evaluated in the PWN criterion.

### Limitations of the study

A limitation of the present study could be its cross-sectional design, comparing only one year of both evaluations. The analysis of a longer historical data series of external evaluations could identify the trend of compliance of the BFHI hospitals practices with the global and additional criteria of BFHI in Brazil.

Another limitation to be pointed out is the representativeness of the analyzed data. Although the number of Brazilian Baby-Friendly Hospitals from 2010 to 2015 ranged from 298 to 334, only 143 performed self-monitoring in all the years of the period, 113 being submitted to external evaluation in 2015. However, in this evaluation, hospitals from the 27 Brazilian states are represented and the non-punitive purpose of self-monitoring does not suggest differential information bias.

## Conclusions

The self-monitoring system partially fulfilled its role of allowing accredited hospitals to self-assess and improve rates of compliance with BFHI criteria. Future trainings of hospital managers need to address difficulties and identify solutions to improve implementation of Steps 4 and 6, as well as reinforce the self-informative and non-punitive role of the self-monitoring tool.

## Abbreviations

BCode: Brazilian Code of Marketing of Breastmilk Substitutes
BFHI: Baby-Friendly Hospital Initiative
HIV: Human Immunodeficiency Virus
PHC: Primary Health Care
PWN: Parents stay with the Newborn admitted to the neonatal unit
UNICEF: United Nations Children’s Fund
WFC: Woman-Friendly Care
WHO: World Health Organization
